# Supplementary material for: Reprogramming of myeloid cells and their progenitors in patients with non-medullary thyroid carcinoma
Source: Nat Commun. 2022 Oct 18;13:6149. doi: 10.1038/s41467-022-33907-4 (PMC9579179; doi:10.1038/s41467-022-33907-4)
Supplement: Supplementary file 1 — Supplementary Information [file 41467_2022_33907_MOESM1_ESM.pdf]

Supplementary information

Reprogramming of myeloid cells and their progenitors in patients with non-medullary thyroid carcinoma

Katrin Rabold[1,2,3,#], Martijn Zoodsma[4,5,#], Inge Grondman[1], Yunus Kuijpers[4,5], Manita Bremmers[6], Martin Jaeger[1,3], Bowen Zhang[4,5], Willemijn Hobo[7], Han J. Bonenkamp[8], Johannes H.W. de Wilt[8], Marcel J.R. Janssen[9], Lenneke A.M. Cornelissen[2], Ilse C.H. van Engen-van Grunsven[10], Willem J.M. Mulder[11,12,13], Jan W.A. Smit[14], Gosse J. Adema[2], Mihai G. Netea[1,3,15], Yang Li[1,3,4,5], Cheng-Jian Xu[1,3,4,5,§], Romana T. Netea-Maier[14,§,*]

[1] Department of Internal Medicine, Radboud University Medical Center, Nijmegen, The Netherlands
[2] Radiotherapy and OncoImmunology Laboratory, Department of Radiation Oncology, Radboud University Medical Center, Nijmegen, The Netherlands
[3] Radboud Institute for Molecular Life Sciences, Radboud University Medical Center, Nijmegen, Netherlands
[4] Department of Computational Biology for Individualised Infection Medicine, Centre for Individualised Infection Medicine (CiiM), a joint venture between the Helmholtz Centre for Infection Research (HZI) and the Hannover Medical School (MHH), Hannover, Germany
[5] TWINCORE, a joint venture between the Helmholtz Centre for Infection Research, (HZI) and the Hannover Medical School (MHH), Hannover, Germany
[6] Department of Haematology, Radboud University Medical Center, Nijmegen, The Netherlands
[7] Department of Laboratory Medicine, Laboratory of Hematology, Radboud Institute for Molecular Life Sciences, Radboud University Medical Center, Nijmegen, The Netherlands
[8] Department of Surgery, Radboud University Nijmegen Medical Centre, Nijmegen, The Netherlands
[9] Department of Radiology and Nuclear Medicine, Radboud University Nijmegen Medical Centre, Nijmegen, The Netherlands
[10] Department of Pathology, Radboud University Nijmegen Medical Centre, Nijmegen, The Netherlands
[11] Biomedical Engineering and Imaging Institute, Icahn School of Medicine at Mount Sinai, New York
[12] Department of Medical Biochemistry, Amsterdam University Medical Centers, The Netherlands
[13] Department of Biochemical Engineering, Laboratory of Chemical Biology, Eindhoven University of Technology, The Netherlands
[14] Department of Internal Medicine, Division of Endocrinology, Radboud University Medical Center, Nijmegen, The Netherlands
[15] Department of Genomics and Immunoregulation, Life and Medical Sciences Institute, University of Bonn, Bonn, Germany

[*]Corresponding author, e-mail: romana.netea-maier@radboudumc.nl

Contents

Supplementary figures 1-5

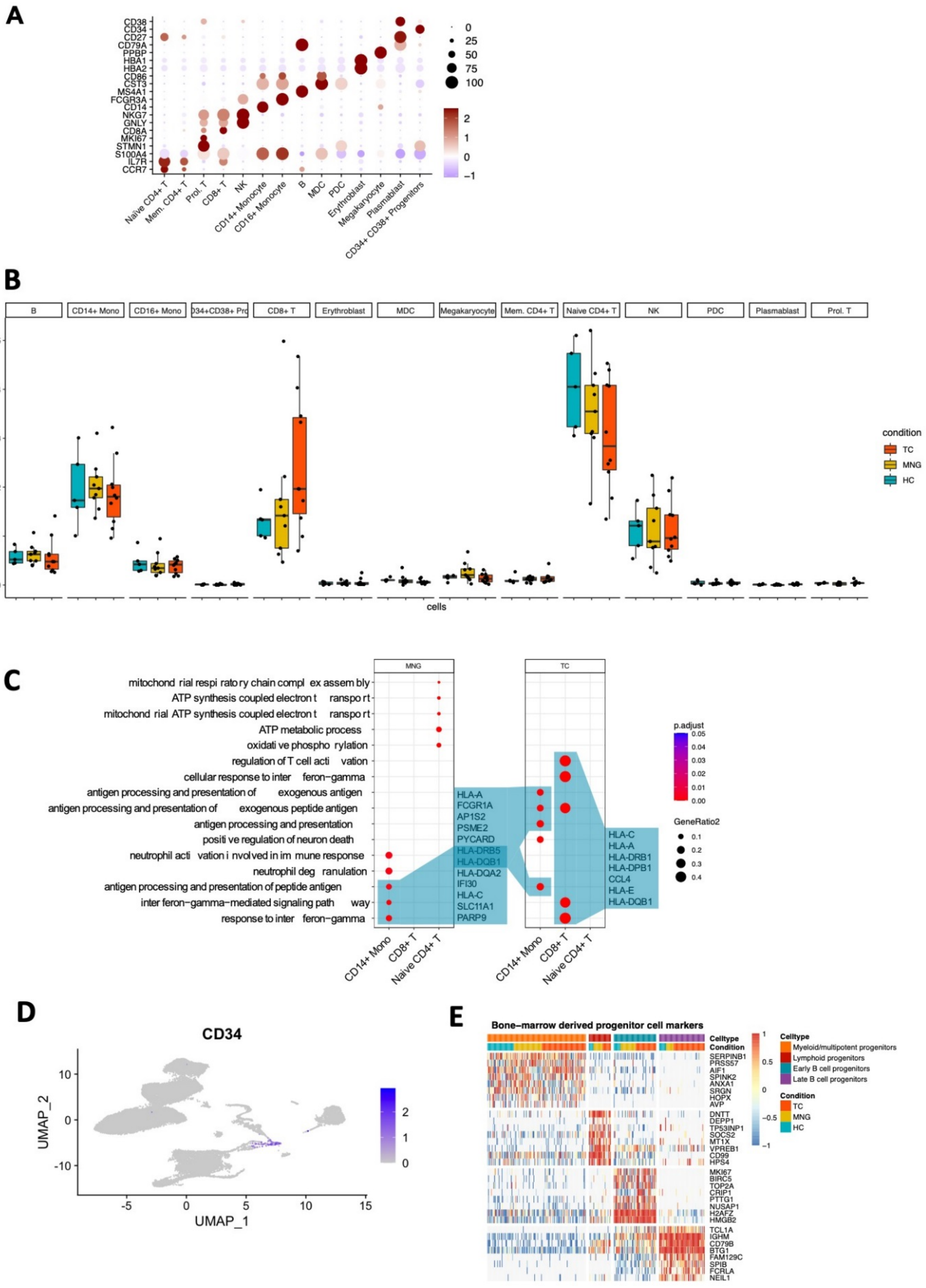
A
CD38
CD34
CD27
CD79A
PPBP
HBA1
HBA2
CD86
CST3
MS4A1
FCGR3A
CD14
NKG7
GNLY
CD8A
MKI67
STMN1
S100A4
IL7R
CCR7
Naive CD4+ T
Mem. CD4+ T
Prol. T
CD8+ T
NK
CD14+ Monocyte
CD16+ Monocyte
B
MDC
PDC
Erythroblast
Megakaryocyte
Plasmablast
CD34+ CD38+ Progenitors
0
25
50
75
100
2
1
0
−1
B
B
CD14+ Mono
CD16+ Mono
CD34+CD38+ Pro
CD8+ T
Erythroblast
MDC
Megakaryocyte
Mem. CD4+ T
Naive CD4+ T
NK
PDC
Plasmablast
Prol. T
cell proportion
cells
condition
TC
MNG
HC
C
MNG
TC
mitochondrial respiratory chain complex assembly
ATP synthesis coupled electron transport
mitochondrial ATP synthesis coupled electron transport
ATP metabolic process
oxidative phosphorylation
regulation of T cell activation
cellular response to interferon−gamma
antigen processing and presentation of exogenous antigen
antigen processing and presentation of exogenous peptide antigen
antigen processing and presentation
positive regulation of neuron death
neutrophil activation involved in immune response
neutrophil degranulation
antigen processing and presentation of peptide antigen
interferon−gamma−mediated signaling pathway
response to interferon−gamma
HLA-A
FCGR1A
AP1S2
PSME2
PYCARD
HLA-DRB5
HLA-DQB1
HLA-DQA2
IFI30
HLA-C
SLC11A1
PARP9
HLA-C
HLA-A
HLA-DRB1
HLA-DPB1
CCL4
HLA-E
HLA-DQB1
CD14+ Mono
CD8+ T
Naive CD4+ T
p.adjust
GeneRatio2
D
CD34
UMAP_2
UMAP_1
E
Bone−marrow derived progenitor cell markers
Celltype
Condition
Myeloid/multipotent progenitors
Lymphoid progenitors
Early B cell progenitors
Late B cell progenitors
TC
MNG
HC
SERPINB1
PRSS57
AIF1
SPINK2
ANXA1
SRGN
HOPX
AVP
DNTT
DEPP1
TP53INP1
SOCS2
MT1X
VPREB1
CD99
HPS4
MKI67
BIRC5
TOP2A
CRIP1
PTTG1
NUSAP1
H2AFZ
HMGB2
TCL1A
IGHM
CD79B
BTG1
FAM129C
SPIB
FCRLA
NEIL1

Supplementary Figure 1.

(A) Dotplot showing the markers used to annotate the PBMC cell populations.

(B) Boxplot of cell proportions within the PBMC populations in HC (n=5), MNG (n=9) and TC (n=10). The center line per boxplot refers to the median, bounds are the 25th and 75th percentiles (interquartile range; IQR). Boxplot whiskers are the smallest and largest values no further than 1.5*IQR from the boxplot bounds.

(C) GO term enrichment analysis of differentially expressed genes in MNG and TC, compared to HC. Highlighted genes are the main DE genes of the corresponding pathways, which partially overlap between the conditions. Over-representation analysis on the significantly differentially expressed genes for each relevant comparison was used to determine significance. P-values were measured using the hypergeometric distribution and adjusted using Benjamini-Hochberg correction.

(D) UMAP projection of 24,470 single cells from 30 samples derived from both PBMCs and BM-MNCs showing CD34 gene expression. Across all cells, there is only one BM-MNC-derived cluster of cells expressing CD34.

(E) Heatmap showing different seurat clusters and their progenitor subtype marker gene expression separated by condition.

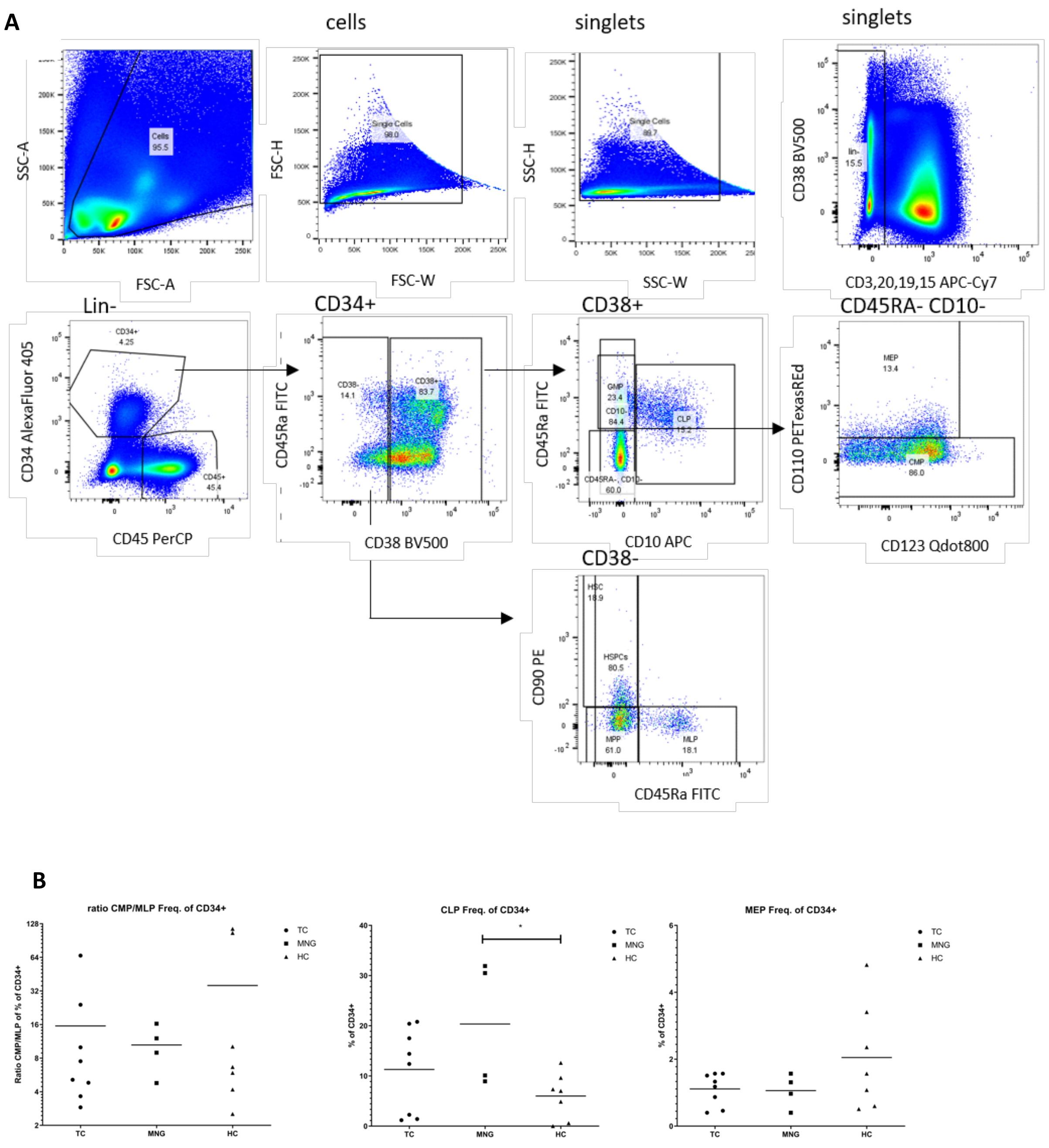

Supplementary Figure 2.

(A) Gating strategy for flow cytometry quantification of bone-marrow derived cell populations.

(B) Flow cytometry quantification of bone-marrow derived cell populations. CMP: common myeloid progenitors. MLP: multi-lymphoid progenitors (cells>singlets>singlets>lin->CD34+>CD38->CD90-CD45RA+ gated cells. CLP: common lymphoid progenitors (cells>singlets>singlets>lin->CD34+>CD38+>CD10+ gated cells). MEP: megakaryocyte-erythroid progenitors (cells>singlets>singlets>lin->CD34+>CD38+>CD45RA-CD10->CD110- gated cells).

Supplementary Figure 3

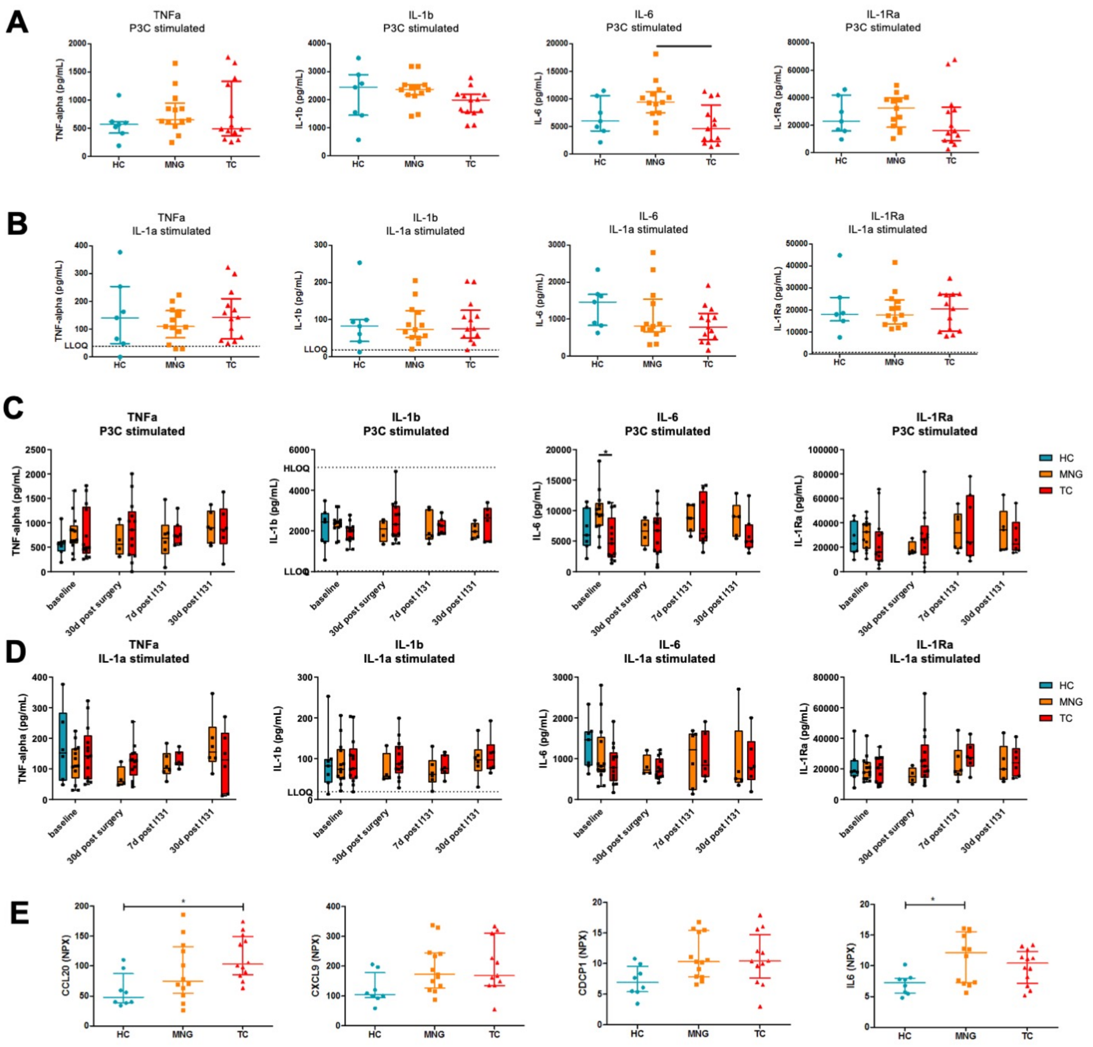

Supplementary Figure 3.

(A) Cytokine release of CD14^{+} CD16^{-} classical monocytes after 24h stimulation with TLR2 ligand P3C at baseline. (HC n=7, MNG n=13, TC n=13) Median with IQR. Corrected for age and sex.

(B) Cytokine release of CD14^{+} CD16^{-} classical monocytes after 24h stimulation with IL-1α at baseline. (HC n=7, MNG n=13, TC n=13) Median with IQR. Corrected for age and sex.

(C) Cytokine release of CD14^{+} CD16^{-} classical monocytes after 24h stimulation with TLR2 ligand P3C during follow-up. (HC n=7; MNG: baseline n=13, 30d post-surgery n=4, 7d post I131 n=6, 30d post I131 n=5; TC: baseline n=13, 30d post-surgery n=13, 7d post I131 n=8, 30d post I131 n=7) p=0.026. Box plot with whiskers from min to max. Corrected for age and sex.

(D) Cytokine release of $CD14^+$ $CD16^-$ classical monocytes after 24h stimulation with IL-1α during follow-up. (HC n=7; MNG: baseline n=13, 30d post-surgery n=4, 7d post I131 n=6, 30d post I131 n=5; TC: baseline n=13, 30d post-surgery n=13, 7d post I131 n=8, 30d post I131 n=7) Box plot with whiskers from min to max. Corrected for age and sex.

(E) Circulating inflammatory markers that are significantly enhanced in TC patients compared to healthy controls (HC n=8, MNG n=13, TC n=12). Median with IQR. IL-6 HC vs MNG p=0.048, CCL20 HC vs TC p=0.015.

HLOQ, higher limit of quantification; LLOQ, lower limit of quantification; *, p <0.05.

Supplementary Figure 4

A

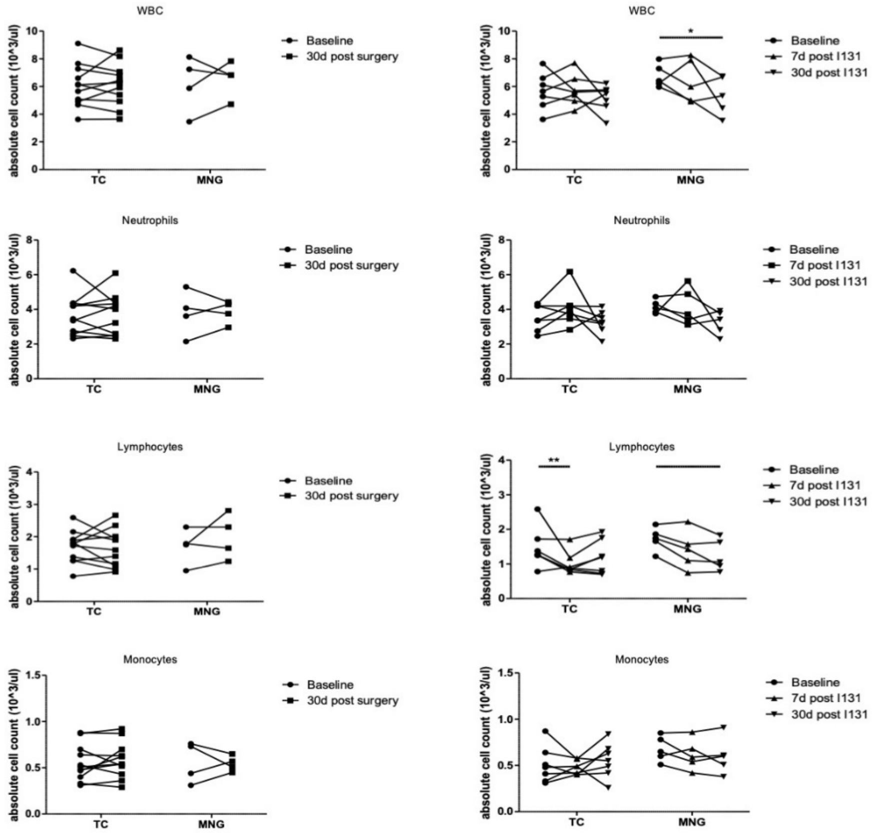

B

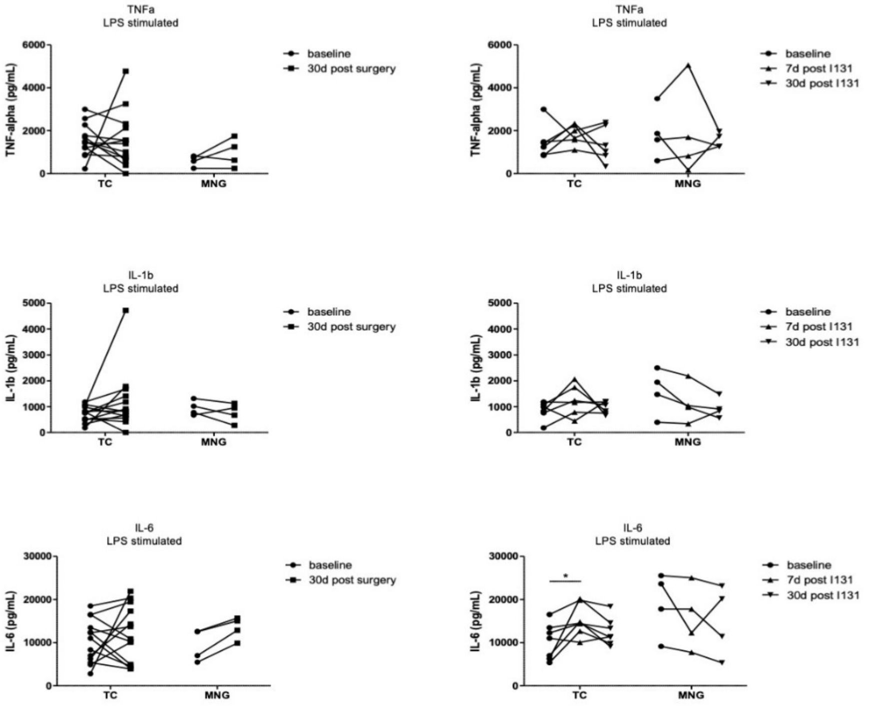

Supplementary Figure 4.

(A) Cell count data analyzed separately for surgery-treated (left- column) and I131-treated samples (right column). Repeated measures two-way ANOVA after exclusion of individuals with missing values.

(B) Analysis of cytokine data upon LPS stimulation using repeated measures two-way ANOVA after exclusion of samples with missing data.

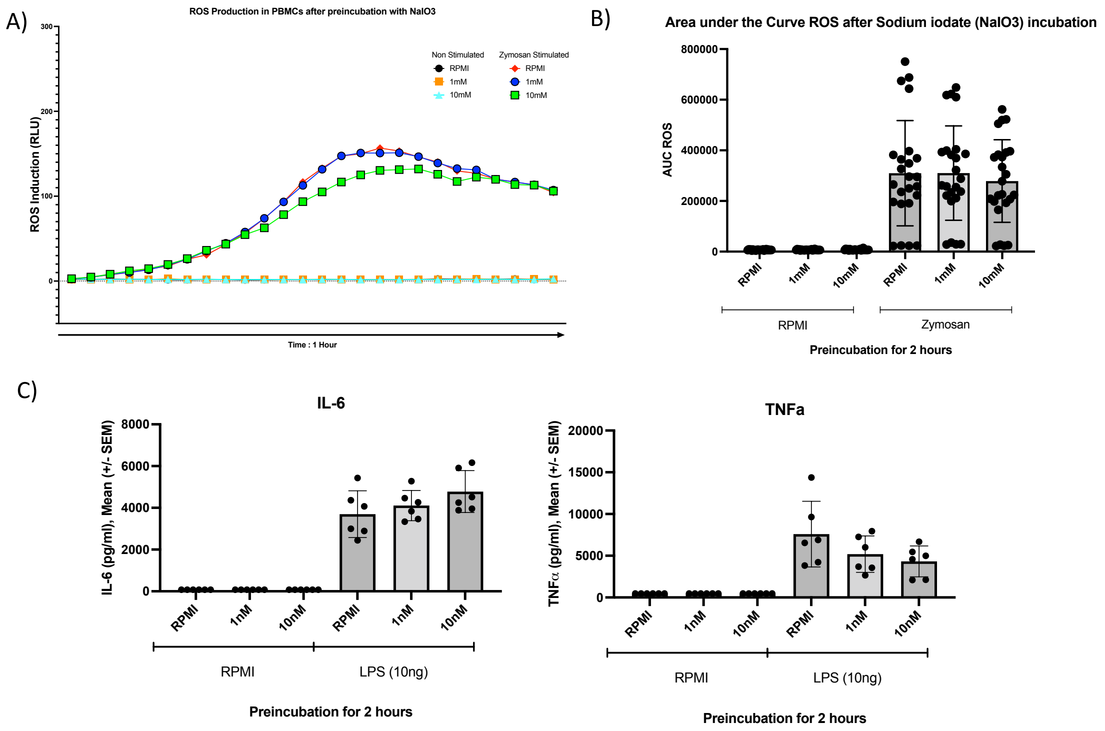

Supplementary Figure 5.

Influence of sodium iodate ($NaIO_3$) on ROS and cytokine production:

(**A)** ROS induction in PBMCs from healthy volunteers (N=3) after preincubation with different concentrations of $NaIO_3$ for two hours and subsequent stimulation with LPS (10ng).

(**B)** Area under the curve ROS.ROS production of healthy volunteers (N=3) after preincubation with different concentrations of $NaIO_3$ for two hours and subsequent stimulation with LPS (10ng). Data are presented as mean values +/- SEM and were analyzed using GraphPad Prism (version 9.20, San Diego, California, USA).

(**C)** Production of TNF-a and IL-6 (N=6) after 2-hour preincubation with $NaIO_3$ followed by stimulation with 10ng/ml LPS for 24 hours. Data are presented as mean values +/- SEM. Data are presented as mean values +/- SEM and were analyzed using GraphPad Prism (version 9.20, San Diego, California, USA).
