# Supplementary material for: Reprogramming of myeloid cells and their progenitors in patients with non-medullary thyroid carcinoma
Source: Nat Commun. 2022 Oct 18;13:6149. doi: 10.1038/s41467-022-33907-4 (PMC9579179; doi:10.1038/s41467-022-33907-4)
Supplement: Supplementary file 3 — Description of Additional Supplementary Files [file 41467_2022_33907_MOESM3_ESM.pdf]

Description of Additional Supplementary Files

File Name: Supplementary Data 1
Description: Raw targeted proteomics dataset used in the manuscript. Exact conditions and sample sizes are specified in the Methods section in the manuscript.

File Name: Supplementary Data 2
Description: Supplementary Data 2: Predicted drug interactions on the significantly differentially expressed genes between TC and HC.
