# Supplementary material for: Reprogramming of myeloid cells and their progenitors in patients with non-medullary thyroid carcinoma
Source: Nat Commun. 2022 Oct 18;13:6149. doi: 10.1038/s41467-022-33907-4 (PMC9579179; doi:10.1038/s41467-022-33907-4)
Supplement: Supplementary file 6 — Reporting Summary [file 41467_2022_33907_MOESM6_ESM.pdf]

nature portfolio

Corresponding author(s): Romana Netea-Maier

Last updated by author(s): June 8th, 2022

Reporting Summary

Nature Portfolio wishes to improve the reproducibility of the work that we publish. This form provides structure for consistency and transparency in reporting. For further information on Nature Portfolio policies, see our Editorial Policies and the Editorial Policy Checklist.

Statistics

For all statistical analyses, confirm that the following items are present in the figure legend, table legend, main text, or Methods section.

n/a	Confirmed	
☐	☒	The exact sample size (*n*) for each experimental group/condition, given as a discrete number and unit of measurement
☐	☒	A statement on whether measurements were taken from distinct samples or whether the same sample was measured repeatedly
☐	☒	The statistical test(s) used AND whether they are one- or two-sided<br>*Only common tests should be described solely by name; describe more complex techniques in the Methods section.*
☐	☒	A description of all covariates tested
☐	☒	A description of any assumptions or corrections, such as tests of normality and adjustment for multiple comparisons
☐	☒	A full description of the statistical parameters including central tendency (e.g. means) or other basic estimates (e.g. regression coefficient) AND variation (e.g. standard deviation) or associated estimates of uncertainty (e.g. confidence intervals)
☐	☒	For null hypothesis testing, the test statistic (e.g. *F*, *t*, *r*) with confidence intervals, effect sizes, degrees of freedom and *P* value noted<br>*Give P values as exact values whenever suitable.*
☒	☐	For Bayesian analysis, information on the choice of priors and Markov chain Monte Carlo settings
☒	☐	For hierarchical and complex designs, identification of the appropriate level for tests and full reporting of outcomes
☒	☐	Estimates of effect sizes (e.g. Cohen's *d*, Pearson's *r*), indicating how they were calculated

Our web collection on statistics for biologists contains articles on many of the points above.

Software and code

Policy information about availability of computer code

Data collection	A detailed description of the data and sample collection can be found in the methods section of the manuscript.<br><br>In synopsis:<br>DNA was isolated from 0.5 mL blood with the ReliaPrep chemistry (Promega) automated on the Freedom Evo Robot (Tecan). Next, genotyping was performed using the GSA-MDv3 array (Infinium, illumina) with a lab-specific clusterfile.<br><br>Peripheral blood mononuclear cells (PBMCs) and and bone marrow mononuclear cells (BM-MNCs) were isolated using optimized density gradient centrifugation. Thereafter, vials were transferred for long-term storage to liquid nitrogen, until further RNA-sequencing analysis. PBMC and MNC samples were prepped for scRNA-seq according to the 10X Genomics Chromium™ Single Cell 3′ v2 RNA sequencing specification. The generated cDNA was used for Illumina next-generation sequencing using a NextSeq500-v2 150 cycle kit with a sequencing depth of 25,000 reads/cell.<br><br>Within the PBMC fraction, classical monocytes were isolated using magnetic cell separation (microbeads, Mylteni biotec protocols) for functional analysis. The freshly isolated peripheral blood classical monocytes were stimulated with different stimuli to assess cytokine production capacity and ROS production, before and after radioactive iodine or surgical treatment .
Data analysis	For data analysis, we employed the statistical language R (version 4.0.3), FlowJo (version 10) and GraphPad Prism (version 8). A detailed description of this analysis can be found in the methods section of the manuscript. All code is made freely available on Github: https://github.com/CiiM-Bioinformatics-group/thyroid-cancer.

For manuscripts utilizing custom algorithms or software that are central to the research but not yet described in published literature, software must be made available to editors and reviewers. We strongly encourage code deposition in a community repository (e.g. GitHub). See the Nature Portfolio guidelines for submitting code & software for further information.

Data

Policy information about availability of data

All manuscripts must include a data availability statement. This statement should provide the following information, where applicable:

- Accession codes, unique identifiers, or web links for publicly available datasets
- A description of any restrictions on data availability
- For clinical datasets or third party data, please ensure that the statement adheres to our policy

The single-cell transcriptomic and genotypic data generated in this study have been deposited at the European Genome-Phenome archive (EGA) under accession number EGAS00001005594. These data are available under restricted access; access can be obtained upon request. Targeted proteomics data generated in this study are provided as Supplementary information. Source data are provided with this paper.

Human research participants

Policy information about studies involving human research participants and Sex and Gender in Research.

Reporting on sex and gender	Our study included patients of both sexes and therefore applies to both sexes. Sexes were self-reported and later confirmed by DNA genotyping. In the study design, sex was included as a covariate to correct for this effect. Sex-specific analyses were not undertaken because of evidence for sex-specific effects in our study.
Population characteristics	The following patients groups were included in this study: patient with newly diagnosed thyroid carcinoma (metastatic or non-metastatic), patients with multinodular goiter receiving surgical or radioactive iodine treatment and healthy controls. General inclusion critiria: at least 18 years old and mentally competent<br><br>Criteria for exclusion were age <18 years, mental incompetence, pregnancy or breastfeeding, inflammatory or infectious diseases or an immunosuppressive status, use of medication interfering with the immune system (e.g. glucocorticosteroids, nonsteroidal anti-inflammatory drugs, cytostatics) diagnosis of clinical thyroid dysfunction at time of inclusion, reduced platelet counts or other conditions associated with an increased risk of bleeding, severe comorbidities such as other active malignancies (except for basal cell carcinoma), serious psychiatric pathology and a self-reported alcohol consumption of >21 units per week
Recruitment	Consecutive patients with multiodular goiter or newly diagnosed thyroid cancer visiting our center between March 2018 and November 2019 were recruited.
Ethics oversight	Ethical approval CMO Arnhem-Nijmegen

Note that full information on the approval of the study protocol must also be provided in the manuscript.

Field-specific reporting

Please select the one below that is the best fit for your research. If you are not sure, read the appropriate sections before making your selection.

☒ Life sciences ☐ Behavioural & social sciences ☐ Ecological, evolutionary & environmental sciences

For a reference copy of the document with all sections, see nature.com/documents/nr-reporting-summary-flat.pdf

Life sciences study design

All studies must disclose on these points even when the disclosure is negative.

Sample size	No statistical tests were undertaken to determine the sample size a priori, and sample sizes were limited by clinical availability. Due to the invasive investigations, the risks and burden on participants was carefully taken into account. Sample size of the current study was deemed sufficient to detect effects statistically.
Data exclusions	No data were excluded in any of the analysis. Of note, for some of the immunological assays performed, not enough biological material was available for some of the patients, and subsequently the final number of tests may differ for the various assessments<br>Data removed for quality control reasons are stated in the Methods section.
Replication	Cytokine assays were performed on duplicates, ROS assays on triplicates. Reported results were consistent across these biological replicates. To reduce inter-assay variation, cytokine assays and proteomics analysis were performed in one batch. To control for inter-plate variation, bridging samples were taken along for cytokine assays and protemoics analysis. Variability between plates was minimal. Flow cytometry validation was used to validate the single-cell RNA sequencing cell population frequencies.
Randomization	This study contained healthy individuals, multi-nodal goiter patients and thyroid cancer patients. There was no randomization between these

Randomization	groups. During inclusion we aimed to distribute, age, gender and BMI between the three groups as comparable as good as practically possible and corrected for age and gender as stated in the manuscript.
Blinding	All experiments were conducted in an unblinded way since the investigators were involved in the planning, execution and analyses of the current study.

Reporting for specific materials, systems and methods

We require information from authors about some types of materials, experimental systems and methods used in many studies. Here, indicate whether each material, system or method listed is relevant to your study. If you are not sure if a list item applies to your research, read the appropriate section before selecting a response.

Materials & experimental systems

n/a	Involved in the study	
☐	☒	Antibodies
☐	☒	Eukaryotic cell lines
☒	☐	Palaeontology and archaeology
☒	☐	Animals and other organisms
☐	☒	Clinical data
☒	☐	Dual use research of concern

Methods

n/a	Involved in the study	
☒	☐	ChIP-seq
☐	☒	Flow cytometry
☒	☐	MRI-based neuroimaging

Antibodies

Antibodies used	Name article number firma Lot<br>FITC anti-human CD45RA Antibody 304105 Biolegend B242180<br>PerCP anti-human CD45 Antibody 304025 Biolegend B242998<br>Brilliant Violet 785™ anti-human CD123 Antibody 306031 Biolegend B253033<br>APC/Cyanine7 anti-human CD3 Antibody 317341 Biolegend B274567<br>APC/Cy7 anti-human CD20 Antibody 302313 Biolegend B275534<br>APC/Cyanine7 anti-human CD19 Antibody 363009 Biolegend B276796<br>APC/Fire™ 750 anti-human CD15 (SSEA-1) Antibody 323041 Biolegend B283027<br>Brilliant Violet 421™ anti-human CD34 Antibody 343609 Biolegend<br>APC anti-human CD10 Antibody 312209 Biolegend<br>PE-CF594 Mouse Anti-Human CD110, clone 1.6.1 562416 BD Biosciences<br>PE anti-human CD90 (Thy1) Antibody 328109 Biolegend<br>Brilliant Violet 510™ anti-human CD38 Antibody 356611 Biolegend
Validation	all antibodies were validated for the species and application as validated by the manufacturer (see manufacturer's website)

Eukaryotic cell lines

Policy information about cell lines and Sex and Gender in Research

Cell line source(s)	TPC-1 is a widely published and well-characterized cell line isolated from a papillary thyroid carcinoma of a female patient.
Authentication	The cell line was not authenticated.
Mycoplasma contamination	All cell lines tested negative for mycoplasma contamination.
Commonly misidentified lines<br>(See ICLAC register)	N/A

Clinical data

Policy information about clinical studies

All manuscripts should comply with the ICMJE guidelines for publication of clinical research and a completed CONSORT checklist must be included with all submissions.

Clinical trial registration	ClinicalTrials.gov identifier: NCT03397238
Study protocol	The study was approved by the local Ethics Committee (Ethical approval CMO Arnhem-Nijmegen 2017-3628). The complete study protocol is available from the corresponding author, [R.M.], upon reasonable request. Study information is also registered at ClinicalTrials.gov (NCT03397238).
Data collection	This study includes samples of subjects who participated in the observational trial "Myeloid Cell Reprogramming in Thyroid Carcinoma" between March 2018 and November 2019.

Outcomes	Our study is an observational study examining primary the tanscriptional and functional reprogramming of myeloid cells, and secodary the change of functional reprogramming after RAI treatment was assessed.

Flow Cytometry

Plots

Confirm that:

☒ The axis labels state the marker and fluorochrome used (e.g. CD4-FITC).

☒ The axis scales are clearly visible. Include numbers along axes only for bottom left plot of group (a 'group' is an analysis of identical markers).

☒ All plots are contour plots with outliers or pseudocolor plots.

☒ A numerical value for number of cells or percentage (with statistics) is provided.

Methodology

Sample preparation	Bone marrow-derived mononuclear cells were freshly isolated by Ficoll density gradient and cryopreserved until analysis. Cell populations in BM-MNCs were identified with the FACSAria III (Becton Dickinson, Franklin Lakes, NJ, USA). 10x106 cells were washed in PBA (0.5% BSA in PBS) after thawing, blocked with Fc-block (Miltenyi Biotec, Bergisch Gladbach, Germany), stained for 45 min at 4C in the dark with the monoclonal antibodies.
Instrument	Becton Dickinson FACSAria III
Software	BD Diva software was used to collect and FlowJo v10 to analyze the data.
Cell population abundance	cell sorting not employed
Gating strategy	Compensation was performed using single stains with compensation beads. For each sample, at least 500,000 counts were recorded. A gate was previously designed based on forward and side scatter to remove debris from analysis.

☒ Tick this box to confirm that a figure exemplifying the gating strategy is provided in the Supplementary Information.
